# What Varieties in Number Are Found in the Teeth of Mammals

**Published:** 1889-01

**Authors:** G. B. Hays

**Affiliations:** U. of M.


### What Varieties in Number are Found in the Teeth of Mammals?

BY G. B. HAYS, U. OF M.

For conveniences in describing and numbering teeth in the various classes of mammalia, it has been found useful by odontologists to adopt a standard of comparison, a dental formula, illustrated by some class as typical and conformed to, more or less by all others. This typical dentition so far as numbers go is expressed by i. $\frac{3}{3}$ c. $\frac{1}{1}$ p. m. $\frac{4}{4}$ m. $\frac{3}{3}$

From this the number varies as widely as do the habits of the different classes of mammals and the uses to which the teeth are subservient. In many cases age is also a varying factor in determining the presence or absence of deciduous teeth.

Based on such a comparison of teeth, the lowest class is the Edentata, which includes the Sloths, Armadillos and Ant-Eaters. There is usually in this class an entire absence of incisors, and although a few have some upper incisors, the centrals are always lacking. The average number is twenty-eight.

In the Cetacea the teeth are usually closely similar in size and appearance when present in large numbers. But many variations are found. The Porpoise developes from 80 to 90. The Dolphin as many as 200.

In the sperm whale teeth are numerous in the lower jaw, while only a few are found in the upper, and these often stunted or buried in the dense gum.

In the Narwhal there are but two, of which one is never erupted from the jaw.

It may be said of the foregoing classes that the rule is a monophyodont dentition, homodont in character and varying in number from one or two teeth to 200.

In the Ungulata and following classes, there is a diphyodont dentition, the deciduous teeth being found at all stages of perfection, from the merest rudiments to the full dentition.

Some like the horse have the full number. The rhinoceros lacks the cuspids and the third incisor above and below.

Of the even-toed ungulata the pig having forty-four teeth is a fair example. The hollow-horned ruminants, including the sheep, oxen, and antelopes, with most of the solid-horned ruminants have the formula i. $\frac{0}{3}$ c. $\frac{0}{1}$ p. m. $\frac{3}{3}$ m. $\frac{3}{3}$.

The camel may be noted as having peculiarities in incisors and molars, there being only one incisor above to antagonize with three below and three pre molars above against two below.

The dentition of the proboscidea may be illustrated by the elephant the only extant member of a large and widely distributed genera. It has in all two upper incisors or tusks and presents a singular method of succession in its molars which replace each other from behind-forwards. Of these there are six on each side of the jaws but only one or parts of two exposed at one time.

The incisors of the rodents are usually two in number, exception being found in hares and rabbits which have four above. The molar teeth of this class are few usually but vary from three to six.

The carnivora are quite regular in the *number* of teeth the greatest variation being in size and form. The three incisors and canine are almost uniform throughout the order. The dog presents the full dentition with the exception of one upper molar. The cat family is strikingly uniform in the formula i. $\frac{3}{3}$ c. $\frac{1}{1}$ p.m. $\frac{3}{2}$ m. $\frac{1}{1}$.

The dentition of the seals is said to be less highly specialized than that of other carnivora, approximating to that of homodont cetaceans.

The common English hedgehog fairly represents the insectivora with i. $\frac{3}{2}$ c. $\frac{0}{0}$ p. m. $\frac{4}{3}$ m. $\frac{3}{3}$.

The teeth of the primates, which order embraces man and the monkeys, are alike in *number*, 32, with the exception of monkeys of the old world which have an additional pre-molar on each side above and below.

Here may well be noted the tendency to obliteration of the third molar from the jaws of the most civilized races. This fact together with its imperfect development, and inferior structure, while it may be a mark between the highly civilized and most barbaric of the human race, must at the same time record a deterioration in the quality of the teeth in general.

On the whole it will be seen that the number of teeth in mammals is widely divergent from any given formula, varying from no teeth at all in the South American ant-eater to the maximum number, two hundred, in the dolphin.

